# Ultrafast charge transfer dynamics in lead sulfide quantum dots probed with resonant Auger spectroscopy at the lead M-edge†

**DOI:** 10.1039/d5ra00479a

**Published:** 2025-05-20

**Authors:** Elin Cartwright, Fredrik O. L. Johansson, Tamara Sloboda, Birgit Kammlander, Andreas Lindblad, Ute B. Cappel

**Affiliations:** a Division of X-ray Photon Science, Department of Physics and Astronomy, Uppsala University Box 516 751 20 Uppsala Sweden elin.cartwright@uu.se fredrik.johansson@physics.uu.se; b Division of Applied Physical Chemistry, Department of Chemistry, KTH Royal Institute of Technology SE-100 44 Stockholm Sweden; c Wallenberg Initiative Materials Science for Sustainability, Department of Physics and Astronomy, Uppsala University 751 20 Uppsala Sweden

## Abstract

PbS quantum dots (QDs) hold significant potential for next-generation photovoltaic and photodetector applications due to their size-dependent electronic properties and strong absorption in the near-infrared region. In this study, we investigate charge transfer dynamics in PbS quantum dots of varying sizes, bulk PbS, and PbI_2_ reference samples using Resonant Auger- (RAS) and Core-Hole Clock Spectroscopy (CHCS). Mapping the Pb M-edge, we capture attosecond-scale electron transfer, using the Pb 3d core-hole lifetime as an internal clock. Our results reveal that PbS bulk samples and larger quantum dots exhibit faster charge transfer rates compared with smaller quantum dots and PbI_2_, which display slower rates. Additionally, by comparing charge transfer times in the Pb MNN and S KLL Auger regions, we demonstrate consistent behavior across different resonant excitation edges, reinforcing our understanding of how quantum dot size and ligand environment influence charge transport. These insights highlight the importance of optimizing QD size and surface chemistry to improve charge transfer efficiency, a critical factor for high-performance energy materials.

## Abstract 2

We investigate charge transfer dynamics in PbS quantum dots of varying sizes, bulk PbS, and PbI_2_ reference samples using Resonant Auger- (RAS) and Core-Hole Clock Spectroscopy (CHCS).

## Graphical abstract

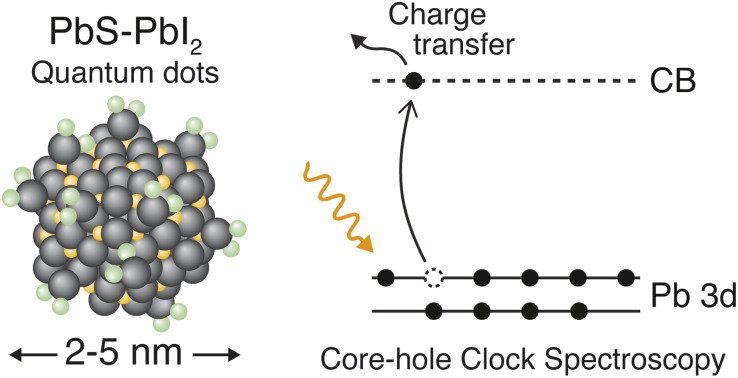

## 1 Introduction

Quantum dots (QDs) have gained significant attention due to their tunable optical and electronic properties.^1^ By decreasing the size of the QD, the band gap increases, leading to absorption and emission that can be tuned to near-infrared and visible wavelengths. When the QD size is equal to or smaller than the Bohr exciton radius (the size of an excited bound electron–hole pair wavefunction^2,3^), the charge carriers become confined within the structural boundaries of the quantum dot. This quantum confinement effect makes QDs suitable for technological applications such as photodetectors, photovoltaics, and thermoelectrics.^4^

One of the semiconductor materials that has been extensively studied in its QD form is lead sulfide (PbS).^5^ PbS is a narrow band gap semiconductor in its bulk form, but exhibits larger band gaps for smaller QDs.^6^ Its strong absorption in the near-infrared region positions PbS QDs as a promising materials candidate for efficient solar cells, photodetectors, and other energy-related technologies.^7–9^ Its large Bohr exciton radius of 18 nm leads to a strong quantum confinement effect even for larger quantum dots,^10^ further motivating PbS as an absorption material. In addition to this, the ease of solution-phase synthesis, such as the hot-injection method used in this study, offers significant advantages in device fabrication and integration.^11^

The QD surface is an important factor in terms of stability and charge transport through the material, and it also impacts the n- *versus* p-type behavior of the material.^8,12^ After synthesis of the QDs, the surface layer can be altered by addition of ligands. These enable formation of air-stable QDs thin films, in which charges can be transported efficiently between QDs.^13,14^ One of the most promising ligands used with PbS QDs so far is lead iodide (PbI_2_) which is used in this investigation.^15–17^ PbS QDs of different kinds have been implemented successfully into solar cell structures, where ligands affect properties and play a large role in carrier mobility.^18,19^

**Fig. 1:**
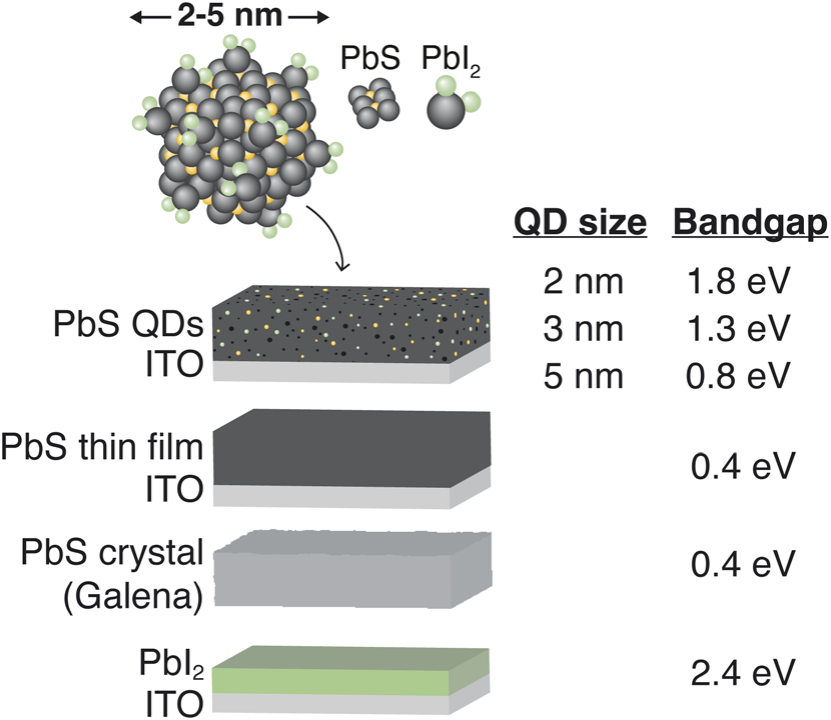
Illustrations of investigated lead-containing samples and their band gap energy.

To optimize their performance in solar cells, it is crucial to understand the charge transfer dynamics at the interfaces of PbS QDs, especially in relation to exciton dissociation and electron/hole transfer rates. Our investigation aims to provide deeper insights into the ultrafast charge transfer mechanisms in QD solids, compared to bulk PbS and PbI_2_ references. By employing Resonant Auger Spectroscopy (RAS) and Core-Hole Clock Spectroscopy (CHCS),^20^ we can study charge transfer dynamics directly from the Pb atom in PbS QDs. By comparing charge transfer times across different quantum dot sizes and reference materials, shown in Fig. 1, we provide a clearer understanding of which factors impact charge transfer in this system. Comprehending these charge transfer processes is essential for improving charge collection efficiency and reducing recombination losses.

**Fig. 2:**
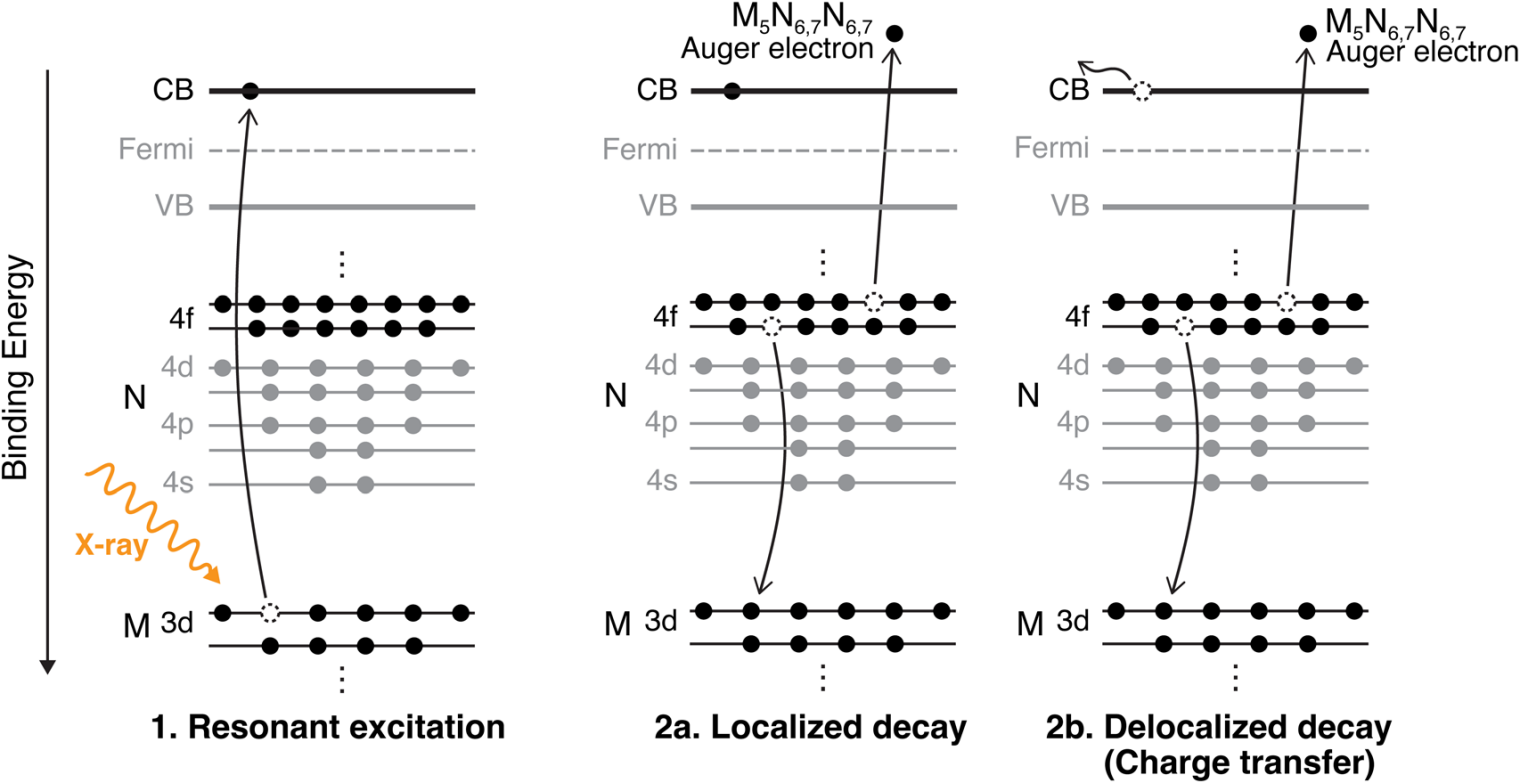
Resonant excitation to the conduction band (CB) followed by either a localized Raman decay (resonantly excited electron remains on the atom) or a delocalized Auger decay (resonantly excited electron tunnels away, indicating charge transfer).

CHCS, a method used within the framework of RAS, enables the study of electron dynamics at atto- and femtosecond timescales by creating a transient core-hole state through X-ray excitation. The lifetime of this core-hole serves as an internal clock, offering precise insights into electron transfer processes critical to charge transport in semiconductor systems. Charge dynamics at interfaces are essential for optimizing materials for energy applications, where efficient transport is a key to device performance. CHCS provides element-specific, site-sensitive information, making it a powerful tool for probing charge transfer mechanisms at crucial junctions in semiconductor devices.^21–24^ Because the Pb 3d core-hole lifetime is only 0.26 fs, CHCS using hard X-rays therefore probes the earliest delocalization step that ultimately governs long-range transport in photovoltaic devices. Hard X-ray excitation does not mimic sunlight, but allows us to resolve the first charge-transfer step on timescales inaccessible to visible-light measurements. As shown in Fig. 2, RAS measurements involve electronic excitation and de-excitation processes that compete within the core-hole lifetime. If the resonantly excited electron remains localized on the Pb atom during de-excitation, the process is categorized as a Raman decay. In contrast, if the excited electron tunnels away from the Pb atom, it is referred to as a delocalized Auger decay, indicating a charge transfer event. The two decay paths can be distinguished when measuring RAS at multiple photon energies over an absorption edge, as the kinetic energy of the emitted electrons will differ. In the delocalized Auger decay, the electron will have the same kinetic energy, regardless of the incident photon energy. In contrast, the electron emitted in the localized (Raman) decay has a constant binding energy, and the measured kinetic energy increases proportionally with the photon energy. The intensity ratio between the two decay channels within the core-hole lifetime provides an estimated charge transfer time, which can be compared across systems to gain deeper insights into the underlying charge transport mechanisms.

We investigate the charge transfer times using CHCS by probing the Pb M-edge, which has not previously been explored. The calculated charge transfer times are compared to those previously published for the S KLL resonance of the same set of samples (except for the PbI_2_ reference sample). In PbS, the Pb 6p level significantly contributes to the conduction band, as shown in the electronic band structure calculations.^25,26^ A resonant transition from the Pb 3d core-level to the conduction band (illustrated in Fig. 2) is expected to be significant owing to dipole selection rules 
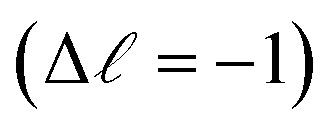
.^27^ By measuring the radiationless decay of this core-excited state we can obtain insight into the Pb contribution to these states and how that impacts charge transfer rates in the system with site-specificity owing to the orbital and elemental/chemical sensitivity of the X-ray core-excitation.

## 2 Experimental details

PbS QDs of sizes 2 nm, 3 nm and 5 nm were synthesized using the hot-injection method and spin-coated on to MgZnO/ITO substrates, as described in our previous publications.^17,28^ Characterization of the QDs was performed using UV-vis/NIR absorption spectroscopy and X-ray diffraction (XRD) to determine QD size and crystal structure, and is reported in our previous publication.^28^ The PbS reference sample was prepared by spin-coating followed by additional surface treatments as previously reported.^17^ The PbI_2_ reference samples were synthesized by depositing PbI_2_ on top of MgZnO/ITO substrates. The naturally occurring lead-sulfide Galena crystal was purchased from Crystal Cave Rocks‡‡https://crystalcaverocks.com. and cleaved immediately before spectroscopic measurements.

Hard X-ray Spectroscopy (HAXPES) and RAS in the hard X-ray regime were measured at the High Kinetic Energy (HIKE) end-station^29^ of the KMC-1 beamline^30^ at the BESSY II synchrotron operated by the Helmholtz-Zentrum Berlin für Materialien und Energie. Equipped with a double crystal monochromator, this end-station reaches a photon flux of 10^11^ photons per s in the photon energy range used in this study. The beamline resolution is about 0.3 eV at the excitation energy used in this work.^29^ Measurements were performed using a Scienta R4000 hemispherical electron energy analyzer with 200 eV pass energy for Auger- and photoelectrons. During all measurements, the samples were positioned close to normal emission to the hemispherical analyzer and at grazing incidence to the incoming light. An estimated experimental resolution was obtained by fitting a Au 4f spectrum with two Voigt functions (Lorentzian and Gaussian convolution), with 0.28 eV (4f_5/2_) and 0.30 eV (4f_7/2_) lifetime broadening.^31^ At the excitation energy 3000 eV, used for sample characterization, this value was found at 0.54 ± 0.03 eV, by the Gaussian contribution to the peak width. At photon energy 2483 eV, the beginning of the RAS map, this value was found at 0.44 ± 0.01 eV.

Resonant Auger 2D maps were acquired by measuring Auger electrons emitted in the Pb M_5_N_6,7_N_6,7_ kinetic energy region around 2180 eV (ref. 32) over a range of photon energies in the Pb M-edge region, step-wise changing the photon energy by 0.5 eV. The photon energy axis was calibrated by measuring Au 4f spectra using the first and third order of X-rays. This procedure was repeated for the photon energy at the beginning of the RAS 2D map and at the end. Spectroscopic data was analyzed in Igor Pro (7.08), using the SPANCF fitting procedures.^33^ Auger peaks were fitted with Voigt functions and Raman peaks were fitted with an asymmetric Doniach–Šunjić^34^ lineshape with a Gaussian convolution. The asymmetry parameter for the Raman peaks was minimal, resulting in a lineshape that closely approximated a Voigt profile. The inelastic background was modeled by a Shirley background.^35^ The line profiles of the Auger and Raman peaks were obtained by fitting them separately where they appear without the other. The parameters for the Auger feature were determined by fitting 5 spectra simultaneously in the high photon energy region. The Lorentzian and Gaussian width parameters were kept fixed between the 5 spectra to find optimum values for the Auger peak. This optimum value was used for fitting the Auger peak for the remaining spectra in the 2D map. The same procedure was carried out for the Raman features, which appear without the Auger peak in the low photon energy region. The positions of Raman peaks R1 and R1′ were fixed to move linearly with the photon energy. Examples of the fitting procedure can be seen in Fig. 4. Additionally, the experimental uncertainties during RAS acquisition were studied by measuring two maps of the same sample (PbS bulk reference) at two different times. The results display a close match in calculated Raman ratio which is shown in the ESI.†

## 3 Results and discussion

### 3.1 Sample characterization

The samples used in this experiment are quantum dot samples of three different sizes (2, 3 and 5 nm) with PbI_2_ surface ligands, a continuous PbS thin film reference, a PbI_2_ thin film reference and a natural PbS crystal (Galena), as displayed in Fig. 1. The quantum dot sizes were determined after synthesis using UV-vis/NIR absorption spectroscopy and XRD to be 2 nm, 3 nm and 5 nm (reported in previous publication^28^), and XRD confirmed the rock-salt crystal structure of the QDs. Core-level HAXPES spectra were recorded of all samples prior to RAS measurements. HAXPES spectra of all samples in the Pb 4f, and S 2p and I 4d core-level binding energy region (including data from previous publication^28^) are displayed in the ESI (Fig. S1).† The Pb 4f core-levels were found at lower binding energies (137.90 eV) for PbS bulk and at higher binding energies for PbI_2_ (138.79 eV), which agrees with previously reported XPS data of these systems.^14^ Pb 4f binding energies for PbS quantum dots were found between these two values, which is expected. All binding energy values of respective samples can be found in the ESI.†

### 3.2 Resonant Auger spectroscopy

**Fig. 3:**
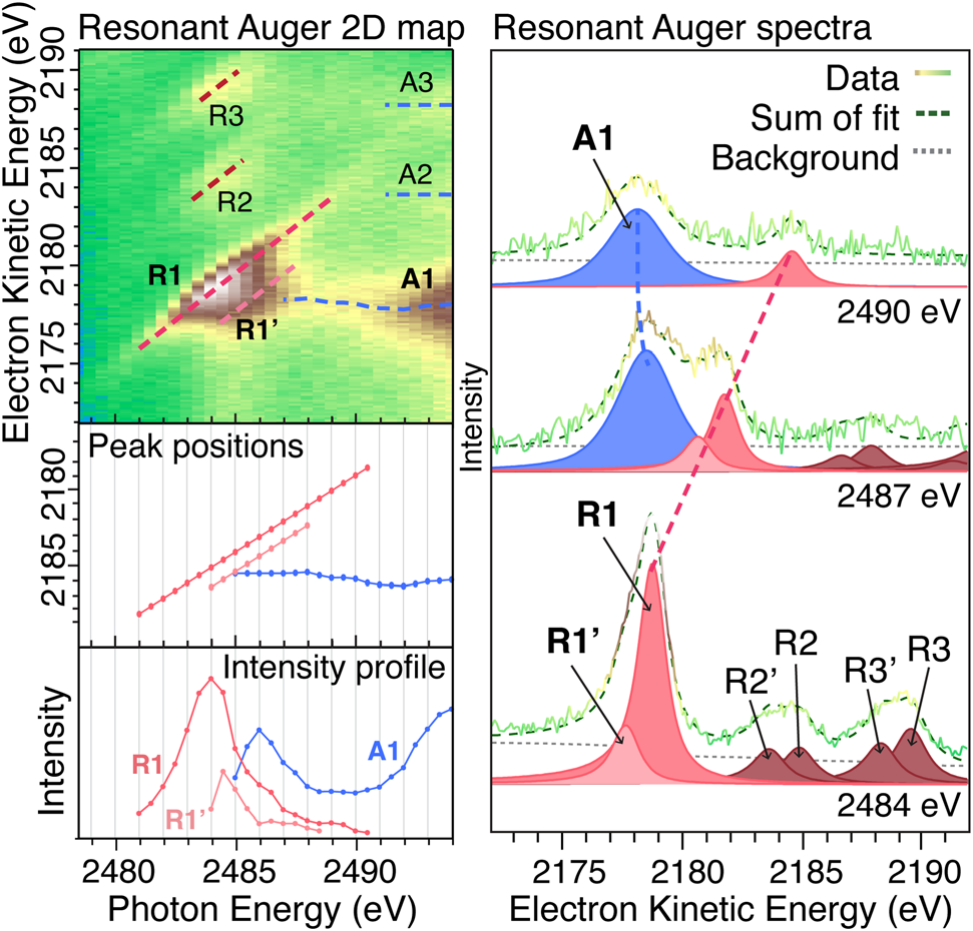
Resonant Auger 2D map in the Pb MNN electron kinetic energy region for the PbS Galena crystal with fitted peak positions and intensity of Raman and Auger features displayed in graphs below. On the right side, curve fittings of resonant Auger spectra are shown for photon energies 2484 eV (bottom), 2487 eV (middle) and 2490 eV (top), illustrating that Raman peaks shift to higher kinetic energy proportionally to the photon energy.

Fig. 3 shows the resonant Auger 2D map for the Galena sample, where the intensity is represented by a color profile. We observe one main Auger line (A1) with highest intensity at kinetic energy around 2178 eV, and two Auger lines with much lower intensity are found at 2184 eV (A2) and 2189 eV (A3), indicating additional transitions with different final states. The corresponding localized decay channels are indicated as R2 and R3, which display lower intensity compared to the main Raman feature (R1). The intensities and positions of the main Auger and Raman features were obtained by curve fitting as outlined in the Experimental details section and are displayed below the 2D map in Fig. 3. Examples of three individual resonant Auger spectra are displayed to the right with the spectral fit included to display the Raman and Auger peaks and their behavior in relation to the photon energy change. The Raman peaks (red) move proportionally towards higher kinetic energies as the photon energy increases.

**Fig. 4:**
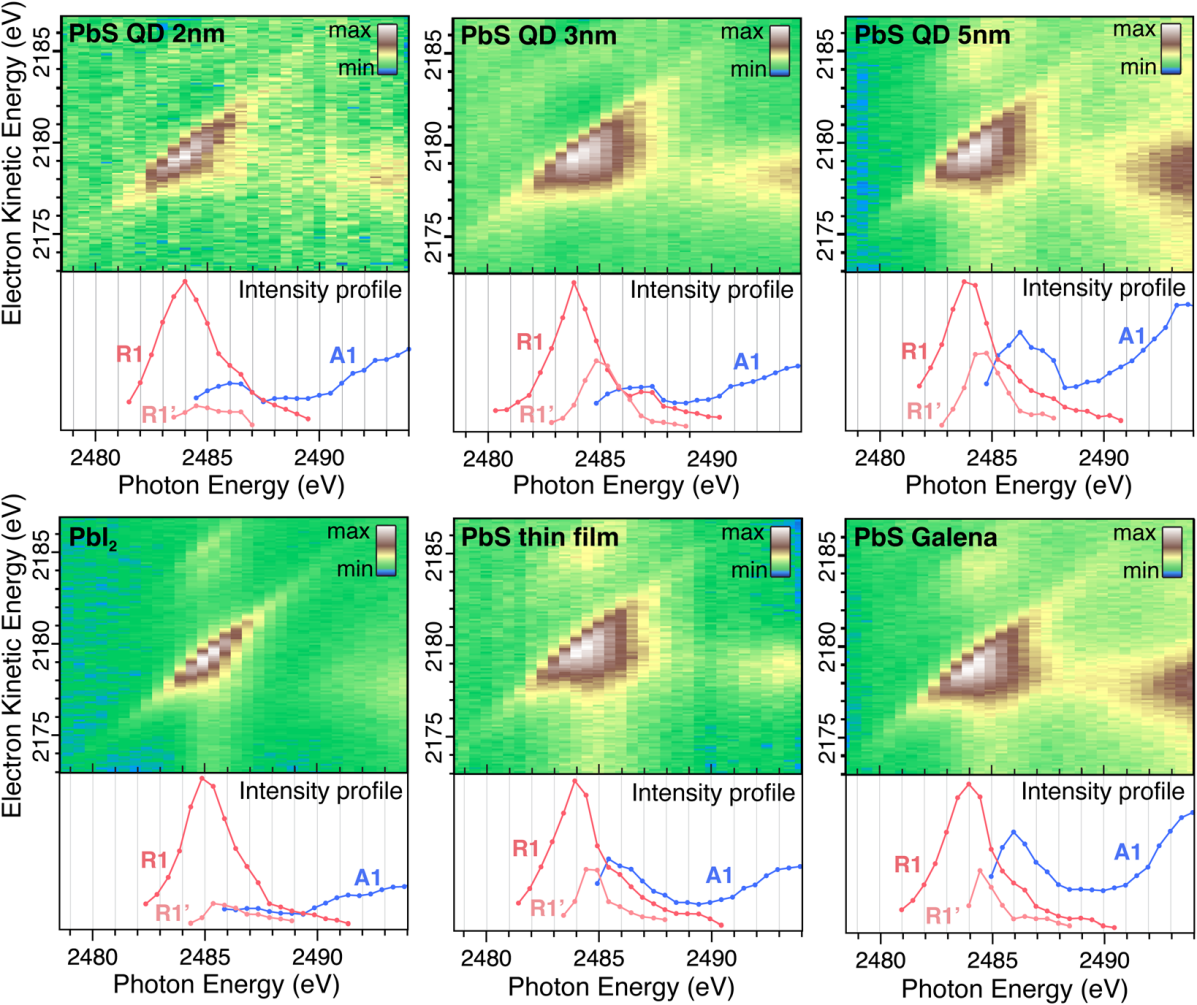
Resonant Auger 2D maps in the Pb MNN electron kinetic energy region for 2 nm, 3 nm and 5 nm PbS QDs, PbI_2_ reference, PbS thin film reference and PbS Galena crystal. Intensity profiles of Raman and Auger features are displayed below each map.

Resonant Auger maps for all samples are shown in Fig. 4 with the intensity profile of the peak components displayed below each 2D map. The intensity profile in the resonance region (photon energies 2483–2488 eV) display notable differences between the samples, indicating differences in the electronic structure surrounding the Pb atom. Specifically, the PbS reference, Galena sample, and larger PbS quantum dots exhibit a pronounced enhancement of the second Raman feature (R1′) at lower kinetic energies relative to the main Raman peak (R1). In contrast, the R1′ intensity remains significantly lower relative to the R1 peak for the PbI_2_ reference sample and the 2 nm QD sample. This suggests that the relative transition rates to the R1 and R1′ states differ between the samples, and the smaller quantum dot is more similar to PbI_2_ and the larger quantum dots are more similar to bulk PbS.

The 5 nm, PbS thin film and Galena have 1.3 eV kinetic energy distance between the R1 and R1′ (1.5 eV, 1.4 eV and 1.2 eV for 3 nm, 2 nm and PbI_2_ respectively). In the electronic structure for PbS calculated in the NaCl structure, two bands in the unoccupied band-structure are found at the *Γ*-point with approximately this separation.^36^ In XANES around the Pb-M_5_ edge it is not possible to discern those two states.^37^ Kim *et al.*^38^ investigated the impact of stoichiometry on the PbS nanoparticle electronic structure – they found that unoccupied electronic states are delocalized and are combinations of mainly Pb atomic orbitals. The distribution of electronic states varies with the size of the PbS quantum dots,^39^ and it also depends on the QD shape and the relative amount of different surfaces since they have different electronic structures.^40^

A shift in the Auger peak towards higher kinetic energies close to the resonance is observed for all samples. This shift occurs at photon energies below the Pb 3d ionization energy and is referred to as Proximity Screening Interaction (PSI).^21^ Similar to post-collision effects, which occur above the ionization threshold,^41^ PSI arises from the screening effect of the excited electron in close proximity to the emitted Auger electron. In the case where the resonantly excited electron has lower excess energy (photon energies of 2491–2493 eV), its delocalization time is extended, allowing it to screen the Auger electron effectively and resulting in a measured increase in kinetic energy. The PSI shift across all samples is between 0.8 eV and 1.3 eV, with no clear trend in the magnitude of the shift observed among different samples.

**Fig. 5:**
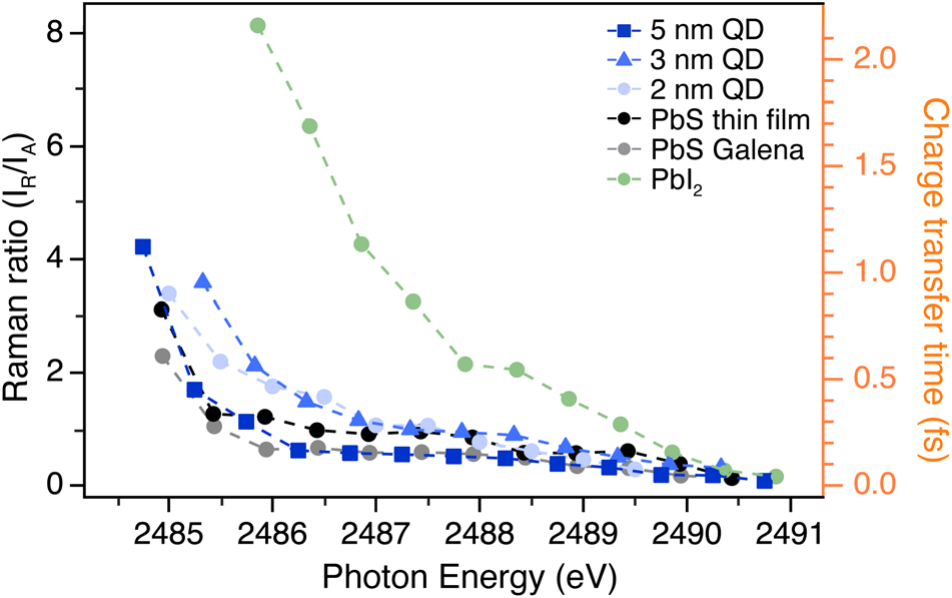
Raman ratios determined from fitted resonant Auger spectra (left axis). Corresponding charge transfer times calculated using eqn (1) (right axis).

The Raman ratio (Σ*I*_R_/Σ*I*_A_) is computed by the intensity ratio between the main Raman (R1 and R1′) and the main Auger (A1) peaks, and is displayed for all samples in Fig. 5 (left axis). The intensities (areas of respective peaks) obtained by curve fitting are compared at each photon energy where both Raman and Auger features are present and reflect the probability of the resonantly excited electron remaining localized on the atom or tunneling away. The largest Raman ratio is found for the PbI_2_ sample, followed by the smaller PbS QDs of 3 nm and 2 nm. The bulk PbS references and the larger PbS QD display lower Raman ratios, both at lower photon energies and higher photon energies. The Raman ratio indicates how efficiently the resonantly excited electron tunnels away from the Pb atom, where a relative increase in the Auger intensity results in a smaller Raman ratio and faster charge transfer.

### 3.3 Core-hole clock spectroscopy

The charge transfer time (electron delocalization time) from the Pb atom following resonant excitation is calculated using the core-hole clock method with eqn (1).^20^ The charge transfer time *τ*_CT_ is obtained by the Raman and Auger intensity relation, multiplied by the core-hole lifetime of the Pb 3d_5/2_ core-hole. The Pb 3d core-hole lifetime *τ*_Pb3d_ is 265.4 ± 27 attoseconds obtained from the lifetime width of 2.48 ± 0.25 eV.^42^ In the same way as for the Raman ratio, only the intensities of the main Auger peak (A1) and the main Raman peaks (R1 and R1′) are used in the calculation.1
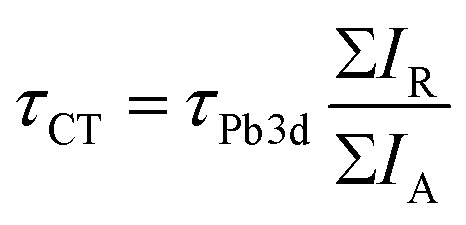


Comparing the charge transfer times across the different samples in Fig. 5, substantial differences are observed among the PbS quantum dots, PbS bulk samples, and the PbI_2_ reference sample. The fastest charge transfer occurs in the PbS bulk samples and the 5 nm quantum dot, while the slowest transfer is seen in the PbI_2_ thin film. The charge transfer times at photon energy 2486 eV and the associated uncertainties are shown for all samples in Table 1.

**Table 1:** Charge transfer times from the core-excited Pb atom at photon energy 2486 eV (data point closest to photon energy 2486 eV)

Sample	*τ*_CT_ (fs)
2 nm	0.47 ± 0.05
3 nm	0.56 ± 0.06
5 nm	0.30 ± 0.03
PbS thin film	0.32 ± 0.04
PbS Galena	0.17 ± 0.02
PbI_2_	2.2 ± 0.2

The largest quantum dots (5 nm) exhibit a charge transfer rate similar to that of the PbS thin film, whereas the 2 nm and 3 nm quantum dots show slower charge transfer rates. In bulk PbS, the electronic states are more continuous and easily accessible, which facilitates more efficient charge mobility and charge transfer. This continuity allows the electrons to interact with a larger network of atoms, reducing localization and enabling faster charge transfer. Although the 5 nm QD is still subject to quantum confinement, the initial step of electron delocalization in the core-excited state occurs on similar timescales as those in the PbS bulk references, owing to the slightly reduced quantum confinement in the 5 nm QDs. By contrast, slower charge transfer is observed in the smaller quantum dots (2–3 nm) which could result from increased quantum confinement which restricts the electron states, leading to a discretization of energy levels that can inhibit efficient electron delocalization and slow down charge transfer rates. Our previous publication on charge transfer from the core-excited sulfur atom shows the same ordering of charge transfer times across the samples as reported here: fastest for PbS references and 5 nm QDs, and slower for 3 nm and 2 nm QDs.^28^ We observe the slowest charge transfer in the PbI_2_ reference sample, likely due to its layered structure and wider band gap, both of which limit the density of available states for electron tunneling.^43,44^

**Fig. 6:**
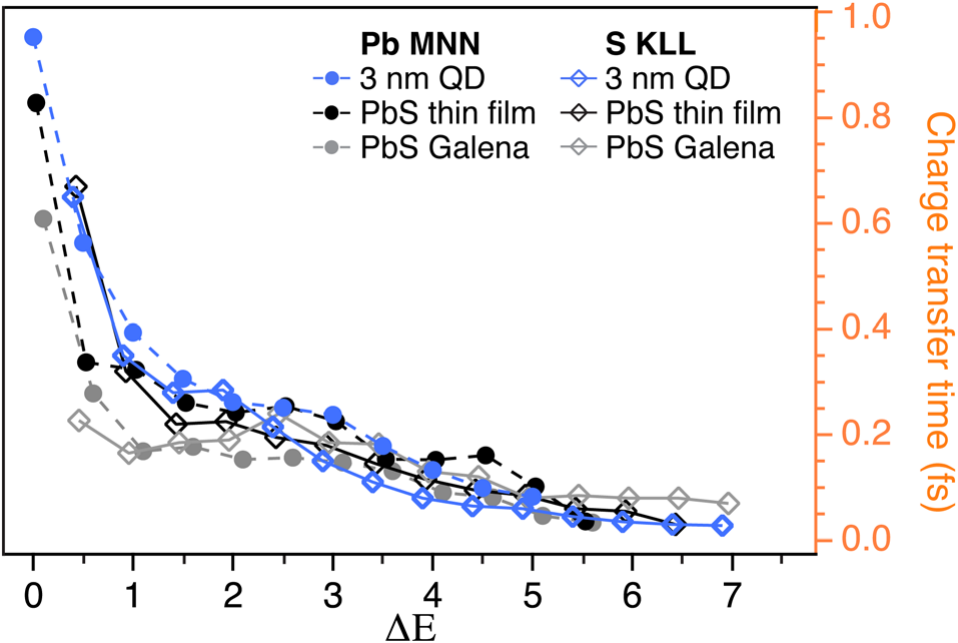
Charge transfer times for PbS bulk references (PbS thin film and Galena crystal) and 3 nm QDs compared for the Pb MNN and S KLL resonant Auger measurements. The *x*-axis (Δ*E*) is the difference in energy between the Raman peak at lower kinetic energy and the Auger peak.

In Fig. 6, the charge transfer times obtained from the previous publication^28^ in the sulfur KLL Auger energy region and the ones obtained here in the Pb MNN region are displayed for the PbS thin film reference, PbS Galena sample and 3 nm QD sample. Here, the *x*-axis represents the energy difference between the Auger peak and the nearest Raman peak (R1′ in the Pb MNN case) at lower kinetic energies. When comparing the calculated charge transfer times in the Pb MNN and S KLL regions, the values are notably similar, indicating that both Pb and S sites contribute to efficient charge transfer. This consistency reflects the strong hybridization of Pb 6p and S 3p orbitals, which facilitates delocalized, isotropic charge transfer across the material.

The opposite trend has been observed in other systems, such as the transition metal dichalcogenide SnS_2_ and PCPDTBT:PCBM bulk heterojunction for organic solar cells. CHCS studies on SnS_2_ revealed significant differences between charge transfer times at the Sn M-edge and S K-edge, driven by the material's layered structure and anisotropic coupling. While charge transfer in SnS_2_ was faster within the layer at the Sn edge, the S edge showed faster interlayer transfer due to closer proximity to neighboring layers.^45,46^ In the case of different mixing ratios of PCPDTBT:PCBM heterojunctions no changes in charge transfer times were seen when making the excitation on the nitrogen atom but strong effects were seen when exciting the different S atoms.^24,47^

## 4 Conclusions

In this study, we explored ultrafast charge transfer dynamics in PbS quantum dots of different sizes, bulk PbS, and PbI_2_ thin film reference samples using CHCS and RAS in the Pb MNN Auger region. Our results show that PbS bulk samples and the largest quantum dots exhibit faster charge transfer rates (0.30 ± 0.03 fs for 5 nm QDs), attributed to reduced quantum confinement effects and increased electronic delocalization. In contrast, slower charge transfer rates in smaller quantum dots (0.47 ± 0.05 fs for 2 nm QDs) suggest the influence of strong quantum confinement. The PbI_2_ thin film displays the slowest charge transfer rate (2.2 ± 0.2 fs), reflecting its reduced density of available states. These atto- to femtoseconds measurements show the direct link between quantum confinement in QDs and ultrafast electron delocalization in the core-excited state.

We also observed that the Pb MNN and S KLL Auger regions yield consistent charge transfer times, suggesting that electronic states on both Pb and S atoms contribute comparably to the charge transfer process in PbS. This is due to the strong hybridization of Pb 6p and S 3p orbitals which facilitates electron delocalization. These findings highlight that size, structural characteristics, and ligand environment critically impact charge transport properties in PbS QDs, with implications for enhancing the performance of PbS-based energy devices.

## Data availability

The data supporting this article, including HAXPES and RAS measurements, are available at the Swedish National Data Source at https://doi.org/10.57804/htfs-3b51.

## Conflicts of interest

There are no conflicts to declare.

## Supplementary Material

RA-015-D5RA00479A-s001
