# Supplementary material for: Ultrafast charge transfer dynamics in lead sulfide quantum dots probed with resonant Auger spectroscopy at the lead M-edge
Source: RSC Adv. 2025 May 20;15(21):16894–900. doi: 10.1039/d5ra00479a (PMC12091093; doi:10.1039/d5ra00479a)
Supplement: RA-015-D5RA00479A-s001 [file RA-015-D5RA00479A-s001.pdf]

Supplementary Information (SI) for RSC Advances.
This journal is © The Royal Society of Chemistry 2025

Supporting Information: Ultrafast charge transfer dynamics in lead sulfide quantum dots probed with resonant Auger spectroscopy at the lead M-edge†

Elin Cartwright,*[a], Fredrik O.L. Johansson,*[a] Tamara Sloboda,[b], Birgit Kammlander,[ab] Andreas Lindblad,[a] and Ute B. Cappel[ac]

[a] *Division of X-ray Photon Science, Department of Physics and Astronomy, Uppsala University, Box 516, 751 20, Uppsala, Sweden.*
[b] *Division of Applied Physical Chemistry, Department of Chemistry, KTH Royal Institute of Technology, SE-100 44 Stockholm, Sweden.*
[c] *Wallenberg Initiative Materials Science for Sustainability, Department of Physics and Astronomy, Uppsala University, 751 20 Uppsala, Sweden*
Email: elin.cartwright@uu.se, fredrik.johansson@physics.uu.se

Sample Characterization

Fig. S1 displays HAXPES spectra of PbS quantum dots, PbS reference samples and PbI_2 reference sample in the Pb 4f, and S2 p and I 4d core level energy region (including data from[1]). Binding energies of respective samples can be found in table S1 below. The binding energy axis is calibrated using the Au 4f core-level of a gold foil attached to the manipulator. The intensity in each spectra is normalized to the total Pb 4f intensity (area under peak from curve fit).

The Pb 4f core level is found at larger binding energies for the quantum dots compared to the PbS bulk reference, and the same behavior is observed for the PbI_2. The shift between PbS bulk and PbI_2 is 0.89 eV, and similar to previously reported values.[2] The PbS Galena crystal and the PbS thin film reference show close values in binding energy for the Pb 4f core level.

Table S1 Binding energies of Pb 4f and S 2p core levels obtained by curve fitting of spectra shown in figure S1.

Sample	B.E. Pb $4f_{7/2}$ (eV)	B.E. S $2p_{3/2}$ (eV)	B.E. I 4d (eV)
Pbs-PbI_2 5 nm	138.06	161.26	49.37
Pbs-PbI_2 3 nm	138.17	161.25	49.40
Pbs-PbI_2 2 nm	138.33	161.32	49.42
Pbs thin film	137.90	161.12	-
PbS Galena	137.88	161.13	-
PbI_2 reference	138.79	-	49.45

The intensity of iodine is largest for the 2 nm quantum dot, which is expected from the difference in surface-to-bulk ratio between the quantum dots of different size. The smallest quantum dot have largest surface-to-bulk ratio which means the relative intensity of Iodine is expected to be largest, with PbI_2 as the surface ligand.

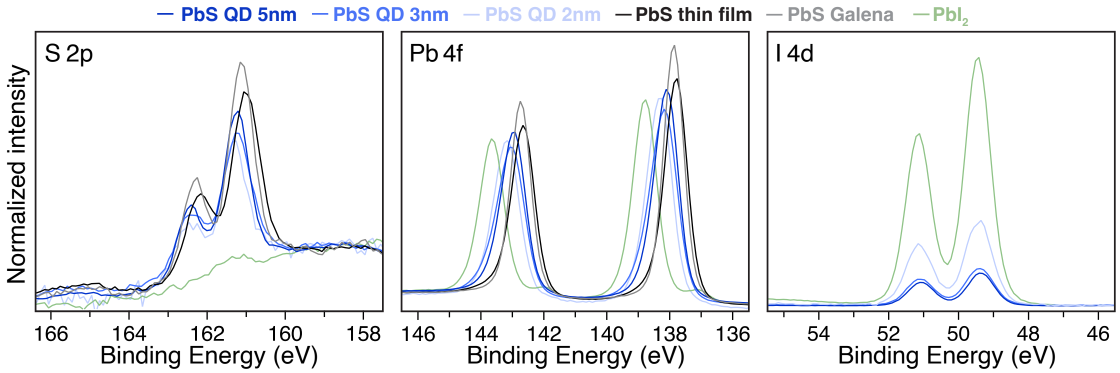

Fig. S1 HAXPES spectra of the Pb 4f, S 2p and I 4d core-levels measured with a 3000 eV excitation energy. The intensity is normalized to the Pb 4f intensity.

Resonant Auger Spectroscopy

The positions of the main Raman (R1 and R1') and Auger (A1) features observed in the Resonant Auger maps are displayed in Fig. S2. The exact positions over the range of photon energies displayed in the figure are obtained by the row-wise curve fitting procedure described in the manuscript.

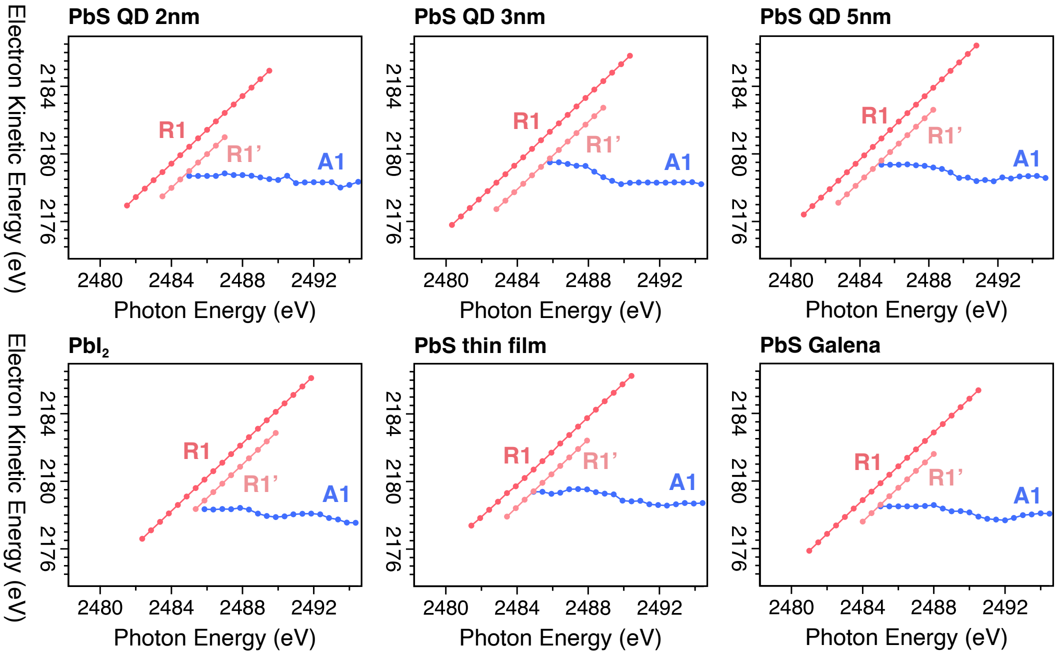

Fig. S2 Peak positions obtained from fitting procedure.

Uncertainty estimation of Raman ratio

To assess experimental errors, two separate RAS maps of the PbS thin film reference were acquired and curve fitted to investigate the spread in Raman ratio. Both maps were acquired under identical experimental conditions at two different times, and the comparison, shown in Fig. S3, demonstrates that the charge transfer times are closely matched. The average difference between data points in the two maps are 11%±3% and the difference is used to estimate errors in the computed charge transfer times.

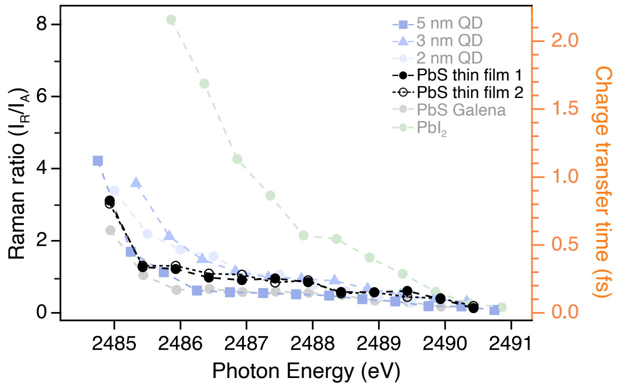

Fig. S3 Raman ratio and charge transfer times compared between two Resonant Auger maps taken of the same sample, PbS thin film reference, at two different times.

Notes and references

1 T. Sloboda, F. O. Johansson, B. Kammlander, E. Berggren, S. Svanström, A. G. Fernández, A. Lindblad and U. B. Cappel, *RSC advances*, 2022, **12**, 31671–31679.

2 J. H. Heo, M. H. Jang, M. H. Lee, M. S. You, S.-W. Kim, J.-J. Lee and S. H. Im, *RSC Advances*, 2017, **7**, 3072–3077.
